# Role of “old” pharmacological agents in the treatment of Cushing’s syndrome

**DOI:** 10.1007/s40618-016-0462-4

**Published:** 2016-04-16

**Authors:** A. G. Ambrogio, F. Cavagnini

**Affiliations:** 1 Department of Clinical Sciences and Community Health, University of Milan, Milan, Italy; 2 Neuroendocrinology Research Laboratory, IRCCS Istituto Auxologico Italiano, Milan, Italy

**Keywords:** Medical therapy, Efficacy, Side effects, Cushing’s syndrome, Cushing’s disease, Adrenal blocking agents, Pasireotide, Cabergoline, Mifepristone

## Abstract

Despite recent advances in the management of endogenous Cushing’s syndrome (CS), its treatment remains a challenge. When surgery has been unsuccessful or unfeasible as well in case of recurrence, the “old” pharmacological agents represent an important alternative for both ACTH-dependent and independent hypercortisolism. Especially in the latter, the advent of novel molecules directly targeting ACTH secretion has not outweighed the “old” drugs, which continue to be largely employed and have recently undergone a reappraisal. This review provides a survey of the “old” pharmacological agents in the treatment of CS.

## Introduction

Endogenous Cushing’s syndrome (CS) is a rare endocrine disorder caused by excess cortisol production driven by a pituitary or extrapituitary ACTH-secreting tumour or directly arising from a primary adrenal lesion. The disease is burdened with high morbidity and mortality [1] and thus prompt cure is mandatory. Surgery remains the cornerstone of treatment regardless of the aetiology of hypercortisolism. In patients in whom surgery was unsuccessful or relapse occurred, repeat surgery, radiotherapy and bilateral adrenalectomy are viable options, though associated with significant side effects. In these cases, medical therapy represents an effective alternative. However, even with the advent of innovative therapies, as drugs directly targeting ACTH secretion for Cushing’s disease (CD), medical treatment of endogenous hypercortisolism is far from being resolving.

Recently, an Endocrine Society Clinical Practice Guideline for the treatment of CS has been published [2], the state of the art of medical therapy in CD extensively reviewed [3] and the use of adrenal blocking agents and glucocorticoid receptor blockers in hypercortisolemic states reappraised [4, 5]. As matters stand, “old” agents continue to be used in clinical practice and have been subject to a reappraisal on large series of patients in order to better define their pharmacological profile. According to their site of action, drugs for CS are classically subdivided into adrenal blocking agents, drugs targeting ACTH secretion and glucocorticoid receptor antagonists. This review will define the current standing of “old” drugs in the treatment of endogenous hypercortisolism.

## Adrenal blocking agents

### Ketoconazole

This antifungal agent has been used off-label in CS since the 1980s. Ketoconazole exerts its effect through interference with enzymes involved in the conversion of cholesterol to cortisol [6]. A further effect at hypothalamo–pituitary level had been reported [7, 8] but not confirmed in later studies. To date, there are no prospective clinical studies on ketoconazole in CS and available data is drawn from retrospective analyses. One of the earliest series was published in 1991 and reported a 93 % response rate in twenty-eight patients with CD [9]. A marked reduction of urinary free cortisol (UFC) levels took place soon after treatment and persisted throughout ketoconazole administration. A parallel improvement, in some cases even disappearance, of clinical signs such as diabetes mellitus, hypertension and hypokalaemia, together with restoration of well-being, was reported. In this series, patients had been submitted to radiotherapy prior to ketoconazole treatment which might account for the brilliant results. Most recently, a French multicenter study reviewed data from two hundred patients with CD and concluded that ketoconazole represents a highly effective drug to control hypercortisolism [10]. In fact, nearly 50 % of patients achieved normalization of UFC (average dose 600 mg ketoconazole q.d.) and an additional 25 % displayed a decrease of UFC by at least 50 %. Preoperative treatment allowed improvement in signs such as hypertension, hypokalaemia and diabetes and thus may have reduced surgical risk. Secondary failure was observed in less than 7 % of patients but some 20 % had to interrupt ketoconazole due to adverse effects, mostly hepatic or gastrointestinal. No fatal hepatitis was reported while both mild (<5-fold normal values) and major (>5-fold normal values) increases in liver enzymes occurred but proved transient in the great majority of patients. Of note, adrenal insufficiency occurred in 5 % of patients at doses ranging from 400 to 1200 mg ketoconazole q.d. The results of the French study are quite similar to those collected in the English literature, showing that 60 % of treated patients achieved UFC normalization [4].

Ketoconazole represents the only drug approved for treatment of CS due to any aetiology by the European Medicines Agency. This recent approval will certainly lead to a revival in the use of this “old” drug. Of note, non-racemic ketoconazole, i.e., only the 2S, 4R enantiomer, is currently being tested in CS.

On balance, ketoconazole is a relevant tool for medical treatment of CS, although mostly a temporary measure. Treatment with ketoconazole should be individualised and few weeks of dose adjustment are usually necessary to control hypercortisolism. Effective dosage ranges from 200 to 1200 mg per day and seems to be quite similar among the different forms of endogenous hypercortisolism. Absorption from the gastrointestinal tract is variable and enhanced in acidic environment [11]. Thus, its serum concentrations may be lower in patients with achlorhydria or on antacids, H_2_ antagonists and proton pump inhibitors. Hepatotoxicity is the most common and possibly serious side effect. Although in most cases liver enzyme elevation is mild and resolves after drug withdrawal, close liver enzyme monitoring is necessary. Adrenal insufficiency is a rare development. No adverse effects on electrocardiographic QT interval were registered after long-term administration in patients with CD [12]. Lastly, ketoconazole is contraindicated in pregnancy since embryotoxicity and teratogenicity have been reported in animal studies. Nonetheless, a favourable outcome has been described in a few unplanned pregnancies [13].

### Mitotane (o,p′-DDD)

Mitotane, also known as o,p′-DDD, is an oral cytotoxic agent mainly used in the management of adrenocortical carcinoma. In addition to its adrenolytic effect, mitotane also inhibits multiple steps in adrenal steroid biosynthesis and may possibly exert an inhibitory effect on tumorous corticotroph cells [14]. Unlike the other steroidogenesis inhibitors, mitotane displays a slow onset of action, which limits its use in cases of severe CS, when a prompt therapeutic response is required. Studies dated over 30 years ago reported clinical and biochemical remission in about 80 % of patients with CD treated with low doses of mitotane and pituitary irradiation [15]. However, relapse occurred in 50–60 % of these patients and additional courses of drug or radiation therapy were necessary. A recent retrospective study on 66 patients with CD who had not been treated with radiotherapy reported sustained normalization of UFC in 70 % of patients after approximately 6 months on mitotane (median daily dose 2.6 g) [16]. Mitotane had to be withdrawn due to lack of efficacy in 15 % of patients despite halving of UFC levels, and in 13 % of patients due to adverse effects, e.g., gastrointestinal or neurologic signs. Although mitotane is an adrenolytic, reappearance of hypercortisolism occurred in some patients on average after 1 year of treatment withdrawal. While plasma mitotane concentrations around 14–20 mg/l are needed in adrenal carcinoma, lower concentrations, i.e., ±8 mg/l, appear sufficient to contain excess cortisol secretion in patients with CD. By low daily doses of mitotane, it has been possible to control cortisol secretion for 10 years in a patient with Carney’s complex and Cushing’s syndrome [17]. Mitotane is approved for use in adrenal carcinoma, both secreting and non-secreting, and in severe CS in several countries. In order to establish its efficacy and safety, assessment of free cortisol in urine or serum has to be made [18] since mitotane increases plasma levels of hormone binding proteins, and thus cortisol binding globulin (CBG) [19], causing spurious elevations of total serum cortisol. Mitotane may alter the metabolism of synthetic steroids leading to an increased replacement need [20] whenever adrenal insufficiency and/or male hypogonadism concur. Elevations of cholesterol and triglycerides are also common. Dose-dependent gastrointestinal, neurological, haematological and hepatic unwanted effects may ensue but are usually reversible upon drug reduction or discontinuation. Due to the highly lipophylic structure of the molecule, mitotane may remain stored in the adipose tissue [21] and maintain for long its effects. There are limited data regarding the effect of mitotane on childbearing potential and pregnancy outcome, thus treatment of women who are or who may become pregnant should be undertaken only after careful balance of risks and benefits.

### Metyrapone

Metyrapone is used in the assessment of hypothalamic–pituitary–adrenal function as well as in the treatment of CS. Its availability is however subject to country-specific regulations, e.g., obtainable in the UK and Italy but not all European countries. Metyrapone inhibits the enzyme responsible for the final step of cortisol biosynthesis, i.e., 11-beta-hydroxylase (CYP11B1) [22]. This causes a rapid fall in cortisol levels with accumulation of cortisol precursors and their entry into the synthetic pathways of androgens and aldosterone. For therapeutic purposes, metyrapone is administered orally in doses varying from 250 mg twice or thrice daily up to a maximum of 6 g per day. Quite recently, a large retrospective study re-evaluated 164 patients with different form of CS who received metyrapone monotherapy across 13 centers in UK over the last 25 years [23]. Overall, more than 80 % of patients showed an improvement in levels of circulating cortisol with over 50 % achieving biochemical eucortisolemia, i.e., early morning cortisol value of 331 nmol/l (12 μg/dl) and mean serum cortisol day-curve of 150–300 nmol/l (10.9 μg/dl). Side effects, mostly mild gastrointestinal upset, occurred in 25 % of patients usually within 2 weeks of initiation or dose increase. A limitation of the study is represented by the measurement of serum cortisol as a marker of biochemical control. When UFC was used, in a series totalling twenty-three patients with CD not previously irradiated, metyrapone achieved biochemical control in 57 % of patients and clinical improvement in 46 % [24]. Escapes were recorded in three patients. Side effects with metyrapone are due to accumulation of androgen and mineralocorticoid precursors, resulting in hirsutism, acne, hypertension and oedema. Attention should be payed to the occurrence of hypocortisolism, whose laboratory diagnosis may be challenging due to the interference of increased levels of 11-deoxycortisol in the cortisol immunoassay [25]. Use of liquid chromatography tandem mass spectrometry (LC–MS/MS) assay is therefore recommended. As for pregnancy, some patients with CS have been successfully managed with metyrapone with no teratogenic effect observed on the foetus [26]. However, worsening of gestational hypertension as well as development of preeclampsia seems to be more likely with this therapy.

LCI699 is a new inhibitor of 11-beta-hydroxylase (CYP11B1) and 18-hydroxylase (CYP11B2), much like metyrapone. In a preliminary proof-of-concept study on twelve patients with CD, administered at a daily dose of 4–100 mg, LCI699 normalized UFC in eleven [27]: studies on larger series are under way.

### Etomidate

Etomitade is a short-acting intravenous anaesthetic in use since the 1970s with an additional, important cortisol-lowering effect [28]. Indeed, a low non-hypnotic dose of 2.5 mg/h or 0.3 mg/kg/h infused for 36 h was noted to cause a rapid fall of serum cortisol with a sustained effect until the end of the infusion [29]. Adrenal suppression occurs at lower doses compared to hypnotic doses [30] and may persist for several days after infusion. Suppression of adrenal secretion, primarily by inhibition of 11-beta-hydroxylase (CYP11B1) and, to a lesser extent, 17-hydroxylase (CYP17A1) and 20,22 desmolase (CYP11A1) [31], proved useful for rapid reversal of hypercortisolism as has been achieved in patients with severe CS, e.g., uncontained psychosis, sepsis [32]. In fact, a protocol for emergency management of CS has been recently drawn up [33]. Long-term control of hypercortisolism has also been reported in individual cases, most notably a young woman with occult ectopic CS in whom etomidate was administered for over 5 months without significant side effects [34]. In this patient, partial inhibition of steroidogenesis persisted at least 14 days after drug discontinuation, probably due to the lipophilic nature of etomidate and its storage in adipose tissue. The use of etomidate in severe cases of hypercortisolism has recently been reviewed [32].

### Other adrenal blocking agents

Aminoglutethimide, an inhibitor of cholesterol side-chain cleavage, 11-beta-hydroxylase (CYP11B1) and 18-hydroxylase (CYP11B2) [35], and trilostane, an inhibitor of 17-beta-hydroxysteroid dehydrogenase, 3-beta-hydroxysteroid dehydrogenase (3-beta-HSD) and 17-alpha-hydroxylase/17,20-lyase (CYP17) [36], have been used in the past but only occasionally in more recent times in the treatment of CS, because of their unsatisfactory pharmacological profile.

## Drugs targeting ACTH secretion

### Dopaminergic compounds: bromocriptine and cabergoline

Dopaminergic agents have been tested in CD and Nelson’s syndrome starting in the mid 1970s and again in more recent years as novel agonists became available. Bromocriptine, a short-acting dopamine D2 receptor (D2R) agonist, was tested first [37]. Reportedly, CD patients bearing a corticotroph adenomatous hyperplasia of the pituitary intermediate lobe, associated with argyrophil fibres, responded better to the dopaminergic compound compared to patients with a pure corticotroph adenoma of the anterior portion of the gland [38]. This finding, with the attendant hypothesis of two different types of CD with different responsiveness to dopaminergic drugs, could not be confirmed in subsequent studies [39]. In patients with CD, monthly intramuscular injection of 50 mg bromocriptine proved unable to correct the hypercortisolism [40], while daily oral administration of the drug was reported to induce clinical and biochemical improvement in a few patients [41, 42]. A high daily dose of bromocriptine, 35 mg/day or more, up to a maximum of 55 mg/day, was tried in six patients affected by CD and a favourable clinical and biochemical response was recorded in three of them [43]. Escape phenomena were registered in responsive patients [43, 44] and long-term control of hypercortisolism has been described only in a scattering of cases [45–47]. While the use of the “old” dopamine agonist was abandoned, dopaminergic agents staged a comeback with the reappraisal of cabergoline, the long-acting D2R agonist already in use for treatment of prolactin-secreting tumours. The demonstration that D2R are expressed in approximately 80 % of pituitary and ectopic ACTH-secreting tumours, with all D2R positive tumours exhibiting a significant inhibition of ACTH secretion when incubated with cabergoline, established the rationale for its trial in CD. Indeed, 8/20 (40 %) CD patients, all expressing D2R, exhibited a sustained normalization of UFC during a 24 month treatment with up to 7 mg weekly cabergoline. Subsequent clinical studies confirmed these early observations [48]. In a prospective study [49], 5/18 patients with CD normalized midnight serum cortisol (MNSC) or low-dose dexamethasone cortisol suppression (LDCS) or both, with a mean weekly dose of cabergoline of 3.6 mg. In four patients treated for 1 year, sustained normalization of MNSC and LDCS took place in two and three of them, respectively. In the same year, normalization of UFC was reported in 3/12 patients (25 %) with CD after 6 months of cabergoline, at dosages up to 3 mg per week [50]. Likewise, in a retrospective analysis, sustained normalization of UFC was observed in 11/30 patients (36.6 %) with cabergoline doses up to 6 mg per week, with nine of the responsive patients remaining in persistent remission after a mean of 37 months of treatment. Two escapes after 2 and 5 years of a complete response are reported [51]. Effectiveness of cabergoline was recently described in two adolescent boys with CD who normalized their UFC after 4 and 6 months of treatment with 1 and 1.5 mg of cabergoline per week and with remission still present after 17 and 24 months of therapy [52]. Altogether, some eighty patients treated with cabergoline have been reported so far and results, while not brilliant, are encouraging with long-term control of hypercortisolism in around 30 % of patients, in some cases for several years [48–52]. Surveillance is recommended for the risk of cardiac valve regurgitation, anyhow low [53]. Novel cabergoline analogues are being developed aimed at improving cardiovascular safety [54].

### Somatostatin analogues

Research into the therapeutic potential of somatostatin analogues in CS began in the 1970s and is still subject to intensive activity. The first studies demonstrated that somatostatin itself as well as its synthetic somatostatin receptor 2 agonist, i.e., octreotide, inhibited ACTH secretion in Nelson’s syndrome [55] and ectopic CS [56, 57]. Instead, octreotide was ineffective in CD [58–60], possibly a consequence of down-regulation of the corticotroph somatostatin receptor subtype 2 (SSTR2) by glucocorticoids [59, 61]. Indeed, combined treatment with octreotide and ketoconazole proved efficacious in three out of four patients with severe CD [62], but this finding was not replicated in subsequent studies and in five patients with CD in whom the pattern of ACTH and cortisol after a single s.c. administration of 100 µg octreotide was not modified by previous administration of 200 mg mifepristone given p.o. 12 and 6 h before the injection [63]. Recent research attempted to widen the use of octreotide, that binds SSTR2, 3 and 5, in endogenous hypercortisolism and assessed its effect in patients with primary pigmented nodular adrenocortical disease (PPNAD), a form of ACTH-independent hypercortisolism characterized by dense expression of most somatostatin receptors in adrenal nodules. In ten patients, a single s.c. injection of 100 µg of octreotide failed to significantly modify cortisol secretion [64].

The demonstration that SSTR5 is highly expressed in the majority of human corticotroph adenomas and is more resistant to glucocorticoid down-regulation, has led to the development of the novel somatostatin analogues, notably pasireotide that binds SSTR1, 2, 3 and, with maximal affinity, SSTR5 [65]. Indeed, long-term pasireotide administration halved UFC excretion in some 40 % of patients and normalized hormonal levels in about 20 % [66]. Untoward effects were those typical of somatostatin analogues, i.e., gastrointestinal symptoms and gallstones, except for hyperglycaemia-related events, which were experienced by over 70 % of patients. In 2012 the European Medicines Agency has approved pasireotide (Signifor), for the treatment of CD in patients who have failed surgery or where surgery is not an option. In this context it has to be recognised that if the only partial efficacy of the old drugs has stimulated the development of innovative compounds like pasireotide, the equally partial efficacy of the novel agents has renewed the interest for the old drugs and for a greater awareness of their use.

### Temozolomide

Temozolomide (TMZ) is an alkylating agent developed 30 years ago and mainly used as adjuvant treatment for glyomas and, to a lesser extent, malignant neuroendocrine tumours. It exerts its cytotoxic effects through its metabolites that methylate DNA at different positions. Methylation at the O6 position of guanine gives rise to DNA adducts, leading to alterations in subsequent DNA replication and eventual tumorous cell apoptosis. The efficacy of the drug relies on the activity of the repair DNA enzyme O6-methyl-guanine-DNA methyltransferase (MGMT) that can reverse alkylation. There is some experimental evidence that low expression of MGMT may be associated with a better response to TMZ [67]. Along the same line, addition of inhibitors of folate metabolism such as pyrimetamine or methotrexate appears to produce synergistic effects [68]. Ten years ago TMZ has been introduced for the treatment of aggressive pituitary tumours (adenomas and carcinomas). The available experience relies on anecdotal reports or small series of patients. However, a review of the literature clearly indicates that the drug, administered orally at the standard regime doses of 150–200 mg/m^2^/day for 5 days every four weeks, is effective [67]. Indeed, it provides a response rate, in clinical and radiological terms, of around 60 % for ACTH-secreting pituitary tumours, 73 % for prolactinomas and 40 % for non-functioning pituitary tumours. Likewise, in another review of the published cases, TMZ has proved capable of inducing a favourable response in 69 % of pituitary carcinomas [69]. Finally, a recent Italian survey has reported on the effects of treatment in thirty-one patients, twenty-five with pituitary adenoma and six with pituitary carcinoma. The drug was given orally at the initial dose of 150 mg/m^2^/day for 5 days every four weeks, with doubling of the dose from the second month on in the absence of severe toxic effects. Treatment, protracted for a maximum of 12 monthly cycles, confirmed its efficacy with twenty-five patients (80.6 %) showing disease control in contrast to six patients (19.4 %) exhibiting tumour progression. In a median follow-up of 43 months, the 2-year progression-free survival and the disease control duration were 47.7 and 59.1 %, respectively [70]. Thus, TMZ appears to be a viable salvage therapy for aggressive pituitary tumours unresponsive to the conventional treatment options. Whether low expression level of MGMT may predict the therapeutic response remains to be established [71, 72].

## Glucocorticoid receptor antagonists

### Mifepristone

Mifepristone, also known as RU-486, is the only drug currently available with anti-glucocorticoid properties. It is a strong competitive antagonist of type 2 glucocorticoid and progesterone receptors. These effects appear to be dose-dependent and blockade of glucocorticoid receptor (GR) occurs at higher doses than those required to saturate progesterone receptor [73]. Of note, mifepristone has a very long half-life compared with other steroids and a relative binding affinity to GR 4 times higher than that of dexamethasone [74] and 18 times higher than that of cortisol [75]. Clinical efficacy of mifepristone in the treatment of CS was originally demonstrated in 1985 in a patient with ectopic ACTH secretion in whom oral drug administration at increasing doses up to 20 mg/kg reversed clinical features of hypercortisolism [76]. More recently, an open-label prospective trial was carried out in fifty patients with refractory CS of various aetiologies, mainly pituitary-dependent CS (86 %) [77]. A starting dose of 300 mg/day was increased step-wise up to 1200 mg/day depending on clinical efficacy. Amelioration of diabetes mellitus and diastolic blood pressure, i.e., the two primary endpoints of the study, was observed in 60 and 38 % of patients, respectively. Moreover, a post hoc analysis of secondary endpoints including glucose homeostasis, blood pressure, lipid control, weight and body composition, physical appearance, strength, neuropsychological status and quality of life also revealed an overall progressive clinical benefit of treatment in 88 % of patients [78] with no significant association between dose escalation and increase of adverse effects [79]. Although poorly understood, a gender-related difference in drug effectiveness, i.e., a faster response to treatment in males, seemed to be present. Administration of mifepristone has also proved helpful in selected paediatric patients [80].

In 2012 the U.S. Food and Drug Administration has approved the use of mifepristone in patients with endogenous CS and type 2 diabetes mellitus or glucose intolerance who were not candidates for or did not benefit from prior surgery. Long-term data on the efficacy and safety of mifepristone in CS are not yet available.

Adrenal insufficiency, albeit uncommon, and hypokalaemia have been reported as major severe drawbacks of mifepristone use [77]. Since serum cortisol levels remain elevated and may even increase during treatment [81], there are no reliable markers both to monitor the biochemical response and avoid acute adrenal failure. The latter occurrence should be carefully watched since it may not be easily reversible by glucocorticoid administration. Indeed, protracted administration of glucocorticoids after mifepristone withdrawal is necessary to overcome its GR blocking effect [82]. Furthermore, increased cortisol levels can lead to severe hypokalaemia and/or hypertension due to the over-activation of mineralocorticoid receptors [83]. In addition, female patients receiving mifepristone may present vaginal bleedings due to endometrial thickening caused by the anti-progestinic effects of the drug [77]. Lastly, caution should be exercised when mifepristone is administered with drugs that are CYP3A or CYP2C substrates, such as simvastatin, cyclosporine, fentanyl, ciprofloxacin, non-steroidal anti-inflammatory drugs and warfarin, since drug-related toxicity might occur. Altogether, also due to its rapid onset of action, mifepristone may be particularly useful in acute hypercortisolemic crises associated with severe clinical manifestation as psychosis.

## Combination treatments

Combined administration of the compounds mentioned above can help to enhance and hasten the effects of treatment in cases of severe hypercortisolism and to reduce the dosage of the single agents so as to improve their tolerability. In this context, addition of ketoconazole enabled UFC normalization in 6/9 CD patients not fully responsive to cabergoline [50]. This sequence of drug administration has been questioned by other investigators who, in a small series, obtained comparable results using the reverse combination, i.e., adding cabergoline to ketoconazole [84]. Likewise, simultaneous administration of mitotane, metyrapone and ketoconazole in eleven patients with ACTH-dependent CS led to rapid and sustained fall of UFC levels, which made possible a successful “rescue adrenalectomy” in five of them [85]. Along the same line, administration of pasireotide with stepwise addition of cabergoline and ketoconazole when a complete response was not achieved, led to UFC normalization in 15/17 patients with CD [86]. Finally, combination treatment with metyrapone and ketoconazole has proved capable of normalizing UFC in 10/14 patients with ectopic ACTH secretion and in 6/8 patients with adrenocortical carcinoma [87].

## Conclusion

Since the first description by Harvey Cushing in 1932 [88] significant advances have been made in the management of endogenous hypercortisolism. While pituitary surgery remains the first-line therapy, medical treatment for patients not attaining remission or experiencing recurrence still largely rests on agents which have been first tested decades ago. Some of them, like ketoconazole, have never been abandoned from their initial use while others like metyrapone, cabergoline and mifepristone have undergone a recent reappraisal. Until compounds capable of satisfactorily containing the ACTH secretion will be developed, the “old” drugs described above will be of help in the management of these patients (Table 1). Authorization for use in CS in Europe or the US has finally sanctioned the importance of “old” drugs or their derivatives, thereby underscoring their usefulness after years of off-label use.

**Table 1:** “Old” pharmacological agents still used for the treatment of Cushing’s syndrome

Drug	Dose	Main adverse events
**Adrenal blocking agents**		
Ketoconazole	400–1600 mg/day	Reversible liver toxicity, gastrointestinal discomfort, gynaecomastia, hypogonadism in men
Mitotane	500 mg/day to 6 g/day	Gastrointestinal discomfort, liver toxicity, impaired concentration and dizziness, gynaecomastia, cholestasis, hyperlipidemia, prolongation of bleeding time
Metyrapone	500 mg/day to 4 g/day	Hirsutism, acne, hypertension, oedema, gastrointestinal discomfort, dizziness
Etomidate	Bolus of 0.03 mg/kg followed by infusion of 0.1 mg/kg/h	Sedation, nephrotoxicity
**Drugs targeting ACTH secretion**		
Cabergoline	1–7 mg/week	Nausea, postural hypotension, headache, cardiac valvular regurgitation (high doses)
Temozolomide	150–200 mg/m^2^/day for 5 days every four weeks	Myelotoxicity, nausea/vomiting, fatigue
**Glucocorticoid receptor antagonists**		
Mifepristone	300–1200 mg/day	Hypokalemia, worsening of hypertension, endometrial hyperplasia and gastrointestinal discomfort, adrenal function assessment precluded
